# Landscape Ecological Risk Assessment Based on Land Use Change in the Yellow River Basin of Shaanxi, China

**DOI:** 10.3390/ijerph19159547

**Published:** 2022-08-03

**Authors:** Zhiyuan Zhu, Zhikun Mei, Xiyang Xu, Yongzhong Feng, Guangxin Ren

**Affiliations:** 1 College of Agronomy, Northwest A & F University, Xianyang 712100, China; zhuzhiyuan@nwafu.edu.cn (Z.Z.); zyzhu@nwafu.edu.cn (Z.M.); 2020050039@nwafu.edu.cn (X.X.); fengyz@nwsuaf.edu.cn (Y.F.); 2 The Research Center of Recycle Agricultural Engineering and Technology of Shaanxi Province, Xianyang 712100, China

**Keywords:** Yellow River Basin, ecological risk assessment, Shaanxi Province, land use

## Abstract

The Yellow River Basin in Shaanxi (YRBS) has a relatively fragile ecological environment, with severe soil erosion and a high incidence of natural and geological disasters. In this study, a river basin landscape ecological risk assessment model was constructed using landscape ecology principles to investigate the temporal and spatial evolution, as well as the spatial autocorrelation characteristics of landscape ecological risks in the YRBS over a 20-year period. The main findings from the YRBS were that the land use types changed significantly over the span of 20 years, there was spatial heterogeneity of the landscape pattern, and the ecological risk value was positively correlated. The threat of landscape ecological risks in YRBS is easing, but the pressure on the ecological environment is considerable. This study provides theoretical support administrative policies for future ecological risk assessment and protection, restoration measures, and control in the Yellow River Basin of Shaanxi Province.

## 1. Introduction

The Yellow River is the second largest river in China and the sixth largest river in the world; it is considered the mother river of China. Thus, the protection and development of the Yellow River Basin is important for the peace and prosperity of the people [1,2,3]. Presently, with the rapid development of urbanization, the interference of human activities on the natural landscape has increased, and the considerable changes in land cover have led to significant changes in the landscape pattern of the basin [4,5,6]. Therefore, studying the temporal and spatial evolution characteristics of land use types and controlling the overall ecological risk in the watershed is beneficial for prescribing the best methods to counteract landscape degradation based on the different risk levels, and give appropriate management and control suggestions. Sustainable development in the Yellow River Basin has important practical and theoretical significance [7,8,9,10].

A watershed is a comprehensive ecological regional system that connects natural ecosystems (land cover types and water cycles) with socio-economic systems (society and population) [11,12,13]. Owing to more complex, holistic, and special location characteristics, the excessive disturbance of a certain element in the basin ecosystem will inevitably threaten the overall stability. The excessive land use changes by humans leads to fragile regional ecosystems [14,15,16,17]. In this study, we performed landscape ecological risk assessments for landscape changes in land use and analyzed the threat of human activities to regional ecosystems. This new ecological management tool provides us with the theoretical support for policy-making, sustainable development, and ecological environment management in river basin risk management and control [18,19,20]. Therefore, this type of assessment has become the main method that is used by scholars to carry out regional ecosystem assessments and is currently a trending topic in landscape ecology [21,22,23,24].

The history of the development of ecological risk assessments has gone through four stages [25,26,27,28,29,30]: (1) the infancy stage (before the 1980s), based on qualitative analysis, mainly focusing on toxicological research on the impact of pollutants on the environment and humans [31,32,33]; (2) human health risk assessment stage (1980s), where the evaluation method was changed to quantitative and the evaluation process and framework were gradually systematic, focusing mainly on human health and their exposure to chemical pollution [34,35]; (3) ecological risk assessment stage (1990s), where the focus changed from environmental and human health risk assessments to ecological risk assessments, and the relevant standard documents of ecological risk assessment were promulgated by many countries and organizations, trying to transform the human health risk assessment framework to the ecological risk assessment framework. Thus, in 1998, the “Guidelines for Ecological Risk Assessment” were released, pioneering progress in the theory and technology of ecological risk assessments [36,37,38,39]. Finally, (4) the regional, landscape, and watershed ecological risk assessment stage (late 1990s to the present), where ecological risk assessment is combined with the theory of landscape ecology, and the research scale extends from population and ecosystem assessments to regional, landscape, and watershed assessments [40,41,42]. Sources and risk receptors both present a period of comprehensive risk assessment from a single development to a variety of risks, such as urbanization, human activities, meteorological changes, and land use changes and are all considered in ecological risk assessments, which uses spatial analysis tools to build models that are based on multi-scale and multi-factor analyses [43,44,45,46].

Shaanxi Province is in the middle reaches of the Yellow River and approximately 70% of the land area and 80% of the population belong to the Yellow River Basin [47]. It is an education, technology, energy, and equipment manufacturing base and the core area of economic development in China. Shaanxi is in an important regional location and has exceptional ecological functions; however, there are still practical problems such as a fragile ecological environment, shortage of water resources, insufficient carrying capacity, and uncoordinated regional social and economic development in the Yellow River Basin. The hilly and gully area of the Loess Plateau in northern Shaanxi is the main source of silt entering into the Yellow River [48,49,50]. A cumulative total of 2.69 × 10^4^ km^2^ of farmland has been restored to forest and grassland, and 15.7 million mu of desertified land has been treated. The Shaanxi section of the basin accounts for more than 83% of the industrial water and more than 78% of the domestic water in the province. The water resource of YRBS provides only 447 m^3^ of water per capita [51], less than one-fifth of the national average. Therefore, in-depth development of the temporal and spatial evolution of land use and landscape patterns, and landscape ecological risk assessments that are based on the theory of landscape ecology in the YRBS is of great strategic significance to scientifically promote the ecological protection and sustainable development of the Yellow River Basin. This will help rationally allocate and utilize land resources and maintain a balanced state of economic and agricultural development [52,53,54,55].

This study was conducted in the Yellow River Basin in Shaanxi. We aim to provide a scientific basis for the ecological protection of the Yellow River Basin in Shaanxi. First, any law changes regarding the different land use types in the basin from 2000 to 2020 were determined; second, the spatial scale of the landscape pattern was studied, and the changes in the landscape pattern were analyzed from both the landscape and patch-type level. Based on the ecological risk assessment of the regional land use landscape pattern of the area, the temporal and spatial evolution and spatial correlation characteristics of the landscape ecological risk were revealed. Finally, considering the results of landscape ecological risk assessment, corresponding ecological risk management countermeasures are proposed.

## 2. Materials and Methods

### 2.1. Study Area

**Figure 1:**
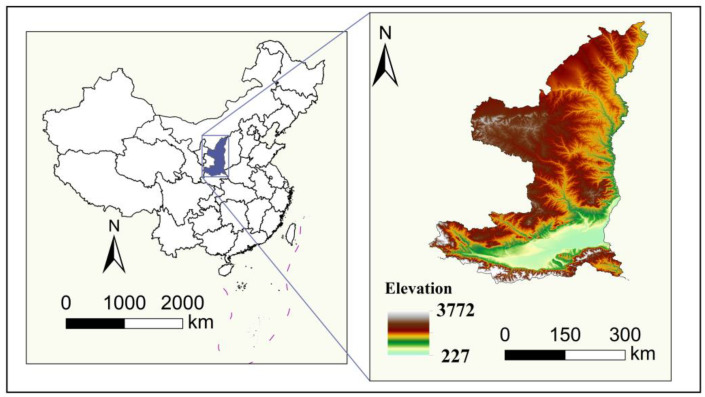
The location of the Yellow River Basin in Shaanxi Province, China.

The Shaanxi section of the Yellow River Basin is in the center of the middle reaches of the Yellow River and passes through the north-central part of Shaanxi Province (Figure 1). It spans approximately 400 km and is connected to Gansu in the west, the Yellow River and Shanxi in the east, Inner Mongolia in the north, and the main beam of the Qinling Mountains in the south, 17.49% of the total area. This section has complex topography, such as undulating mountain ranges, vertical and horizontal rivers, high-lying areas in the north and south and low-lying in the middle, and slopes from west to east. The Shaanxi section of the Yellow River Basin is composed of 79 counties (districts and cities) in 8 cities, namely Yulin, Yan’an, Tongchuan, Baoji, Xianyang, Xi’an, Weinan, and Shangluo, with a population of about 29.15 million, accounting for 75.41% of the total population of the province [56]. It spans two climatic zones, roughly bounded by the Great Wall of China, the north is located in the middle temperate zone and the south is in the warm temperate zone. The natural vegetation is considerably varied, with grasslands and shrubs in the north and forests in the south.

### 2.2. Data Collection and Processing

There were three periods of land use data from Shaanxi Province (shp. Format), namely, 2000, 2010, and 2020, that were used in this study, derived from the GlobeLand 30 surface cover dataset (raster data, with a resolution of 30 m) (http://www.Globallandcover.com/ (accessed on 1 July 2022). The maps of the administrative division of the study area and the water system of the Yellow River Basin were obtained from the Resource and Environmental Science Data Center of the Chinese Academy of Sciences (http://www.resdc.cn (accessed on 1 July 2022)). The vector boundary of the YRBS used the hydrological model in ArcGIS software v10.2 (Esri, Redlands, CA, USA) The watershed delineation tool in ArcSWAT used the automatic watershed delineation command to generate watershed divisions. Based on the national land use classification system and according to the land use characteristics and research purposes of YRBS, the land use types in the study area were divided into eight categories: cultivated land, forest, grassland, shrubland, wetland, water body, artificial surface, and bare land.

### 2.3. Methods

**Figure 2:**
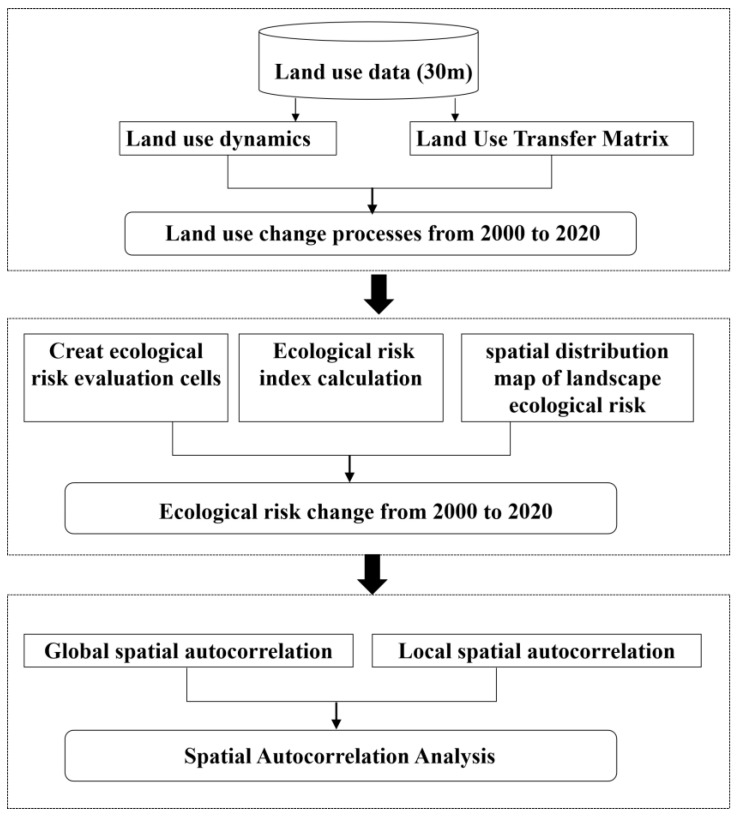
The workflow of the research.

In order to study the temporal and spatial variation characteristics of landscape ecological risk in the YRBS, the workflow is as follows (Figure 2). First, based on land use data in 2000, 2010, and 2020, Land use dynamics and the Land Use Transfer Matrix were used to explore the process of land use change in the YRBS. Then, we evaluated the landscape ecological risk and discuss its temporal and spatial variation characteristics. Finally, the spatial autocorrelation of ecological risk index was analyzed by Moran’s I index and local spatial autocorrelation analysis method.

#### 2.3.1. Land Use Dynamics

The dynamic degree of land use quantitatively expresses the speed of land use change in certain periods, measures the difference in land use change between different regions, and predicts the future trend of land use change in the region. The two land-use dynamic degrees that were used in this study were the single land use *K* and the comprehensive land use *S*. The larger the absolute value of the single land use dynamic degree is, the faster the transformation speed of the land use type. The comprehensive land use dynamic degree indicates the degree of land use change in the study area from a macro perspective, and the larger the dynamic degree is the more severe the degree of change. The specific equations are as follows:

$$K=\frac{U_{m}-U_{n}}{U_{n}}\times \frac{1}{T}\times 100\%$$

$$S=\overset{n}{\underset{ij}{\sum }}\frac{\Delta S_{i-j}}{S_{i}}\times \frac{1}{T}\times 100\%$$

where *K* is the dynamic degree of a certain land use type in the research period; $U_{n}$ and $U_{m}$ are the area (km^2^) of the land use types in the study area at the beginning and end of a certain period, respectively; and *T* is the research time (years). *S* represents the comprehensive land dynamic degree, *S…* is the total area (km^2^) of the *i*-type land use that is converted to other land use types in the *T* period

#### 2.3.2. Land Use Transfer Matrix

For the land use transition matrix, we used the Markon transition probability matrix. The Markon model can not only directly and specifically quantify the structural characteristics between the changes of land use types, but also show the number of transfers between different land types; thus, quantitatively showing the degree of system analysis on the system state and state transfer. The following equation was used:

$$S_{ij}=\left[\begin{array}{c} \begin{array}{c} \begin{array}{ccc} S_{11} & S_{12} & \cdots \\ S_{21} & S_{22} & \cdots \\ \vdots & \vdots & \vdots \\ S_{n1} & S_{n2} & \cdots \end{array}\;\begin{array}{c} S_{1n}\\ S_{2n}\\ \vdots \\ S_{nn} \end{array} \end{array} \end{array}\right]$$

where *S* represents the transition matrix of land use change; *n* is the total number of different land types (*n* = 8); *i* and *j* represent the initial and final land types, respectively, in the study area (*i*, *j* = 1, 2,…, *n*); and *S* is the area of the *i*th land type that is converted to the area of the *j*th land type, the larger the value, the more severe the change, and vice versa.

#### 2.3.3. Landscape Ecological Risk Assessment

**Figure 3:**
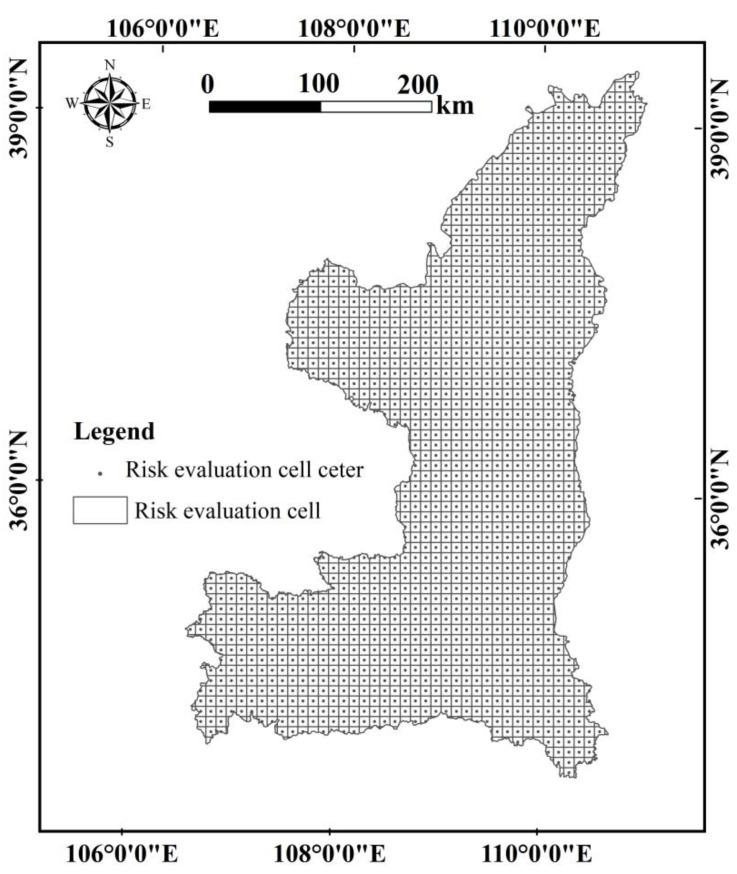
The ecological risk evaluation cells.

According to the characteristics of the area of the YRBS, the moderateness of the data sampling workload and the accuracy of the evaluation unit, the study area is divided into a 10 × 10 km grid size by using the equal-spaced systematic sampling method and the Create Fishnet tool of ArcGIS 10.2 software (Esri, Redlands, CA, USA). A total of 1500 risk cells (as shown in Figure 3). Then, ArcGIS 10.2 software was used to calculate the landscape ecological risk value of each risk area, and the ecological risk index was assigned to the center of each risk area, and then the ordinary Kring interpolation method of spatial interpolation was used to obtain the spatial distribution map of landscape ecological risks in the YRBS. In this way, the landscape ecological risk assessment of the entire watershed can be carried out.

The landscape disturbance index (*E_i_*) and landscape vulnerability index (*F_i_*) were selected in this study for constructing a comprehensive Ecological Risk Index (*ERI*) model for the YRBS. The landscape ecological risk index was calculated as follows:

$$ERI_{i}=\overset{n}{\underset{i=1}{\sum }}\frac{A_{ki}}{A_{k}}\times R_{i}$$

where $ERI_{i}$ is the landscape ecological risk index of the *i*th risk unit, *n* is the number of landscape types, $A_{ki}$ is the area of the *i*th of landscape type in the *k*th risk unit, and $A_{k}$ is the total area of the *k*th risk unit. $R_{i}$ is the landscape loss index of the *i*th landscape type, which is calculated from the landscape disturbance degree and vulnerability index. The equations for each landscape pattern index in the model are shown in Table 1.

Finally, the natural breakpoint method is used to divide the ecological risk into five grades, as shown in Table 2.

#### 2.3.4. Spatial Autocorrelation Analysis

(1) Global spatial autocorrelation

Global spatial autocorrelation analysis was used to measure the agglomeration characteristics of the attribute values in the entire study area, reflecting the approximation of the attribute values of adjacent units. Generally, *Moran’s I* index was used to characterize the degree and significance of spatial autocorrelation of ecological risks in the study area. The equation is as follows:

$$Moran^{’}s\;I=\frac{n\sum_{i}\sum_{j}W_{ij}\left(x_{i}-\bar{x}\right)\left(x_{j}-\bar{x}\right)}{\left(\sum_{i}\sum_{j}w_{ij}\right)\sum_{i}{\left(x_{j}-\bar{x}\right)}^{2}}$$

where $x_{i}$ and $x_{j}$ are the attribute values of the variables in the adjacent units of the region, $W_{ij}$ is the spatial weight matrix, and $\bar{x}$ is the average attribute value. The value range of $Moran^{’}s\;I$ index is (1, 1) which means that the positive correlation of similar spatial proximity transitions to the negative correlation of spatial proximity dissimilarity. When $Moran^{’}s\;I$ > 0, the spatial correlation is positive and the unit attribute value presents spatial clustering characteristics, and the closer the value is to 1, the higher the degree of agglomeration. When $Moran^{’}s\;I$ < 0, the space is negatively correlated, and the unit attribute values show spatially discrete characteristics. When $Moran^{’}s\;I$ = 0, there is no spatial correlation, and the unit attribute values are random with an independent distribution status.

(2) Local spatial autocorrelation

The global spatial autocorrelation represents the overall spatial state of the attribute value and cannot reflect the specific location of the agglomeration or abnormal attribute value in the spatial distribution. In this case, the local autocorrelation method needs to be used for further explanation. Local spatial autocorrelation mainly reveals the heterogeneity of the spatial distribution of local unit attribute values, including all spatial unit attribute values in the study area. Local *Moran’s* *I* (*I_i_*) is used to express the local spatial autocorrelation and it is calculated as follows:

$$I_{i}=\frac{\left(x_{i}-\bar{x}\right)\left[\left(n-1\right)-{\bar{x}}^{2}\right]}{\sum_{j=1}^{n}x_{ij}^{2}\sum_{i=1}^{n}\sum_{j=1}^{n}W_{ij}\left(x_{j}-\bar{x}\right)}$$

where $x_{i}$ and $x_{j}$ are the attribute values of the variable in the adjacent units of the region, $W_{ij}$ is the spatial weight matrix, and $\bar{x}$ is the average attribute value. When *I_i_* ≥ 0, the attribute value of the *i*th unit is similar to the attribute value of the adjacent unit, and the attribute value of the unit presents spatial discrete characteristics, which is a positive spatial correlation. When *I_i_* < 0, the attribute value of the *i*th unit is related to the adjacent unit, the attribute values of the units are quite different, and the unit attribute values show spatial discrete characteristics, which is a negative spatial correlation.

## 3. Results

### 3.1. Land Use Change Processes from 2000 to 2020

#### 3.1.1. Analysis of Land Use Dynamics Change

Using the dynamic degree formula to calculate the three-phase land use data in the Shaanxi Yellow River Basin from 2000 to 2020, we obtained the dynamic degree of each land use type in the study area from 2000 to 2010, 2010 to 2020, and 2000 to 2020 as shown in Table 3.

As shown in Table 3, there are discernable differences in the single dynamic degree of each land use type during the study period, and the overall performance adheres to the following order: artificial surface > shrubland > wetland > bare land > grassland > cultivated land > water body > forest. The type of land cover with the greatest variation in single dynamics is always artificial surfaces. Between 2000 and 2010, the greatest change in the single dynamic degree was that of shrubland and artificial surfaces, which changed by 3.14 and 3.11%, respectively, and the area increased by 159.93 km^2^ and 1072.16 km^2^, respectively. Between 2010 and 2020, except for artificial surfaces and water bodies, the single dynamic degree of the utilization type is in a decreasing state, and the artificial surface is still the land use type with the largest change in single dynamic degree. The area has increased by 1665.78 km^2^ in 10 years, and the dynamic degree is 3.59%. The single dynamic degree of the water bodies initially had a negative change but turned positive. The arable land, grassland, and wetland continued to decrease, and the change rate of grassland accelerated significantly compared to that in 2000–2010, the area decreased by 1232.16 km^2^, and the change rate of arable land and wetland slowed down compared to the previous 10 years. Shrubland showed a significant slowdown with a single dynamic degree of 0.41% and went from a positive change at the beginning to a negative change. In general, artificial surfaces have grown positively throughout the research period from 2000 to 2020. The area increased by 2737.94 km^2^ over 20 years, and the single dynamic degree is at most 4.21%. In 2010, there was rapid economic and social development in China, the pace of urbanization accelerated, anthropogenic interference increased, the change rate of land use types accelerated, and the change rate of man-made surfaces increased faster than that of other land types. In addition, all the remaining land use types showed fluctuating changes except for cultivated land, grassland, and wetlands, which showed a constant decreasing trend over the span of 20 years.

#### 3.1.2. Analysis of Land Use Transfer Change

To further visualize the spatial evolution characteristics and mutual transformation rules of various land cover types in the Yellow River Basin of Shaanxi Province, we used GIS spatial analysis technology and the land use transfer matrix model to analyze the direction and quantity of changes between various land use types.

**Figure 4:**
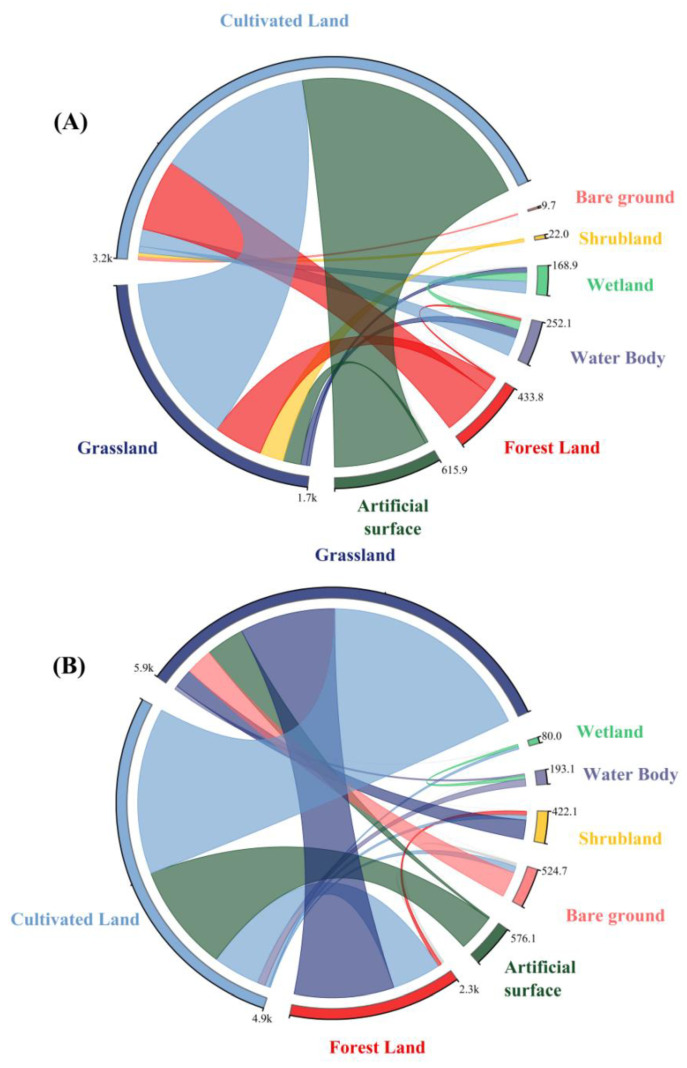
Land use type transfer area (km^2^) matrix of the YRBS from 2000 to 2010 (**A**) and 2010 to 2020 (**B**).

Figure 4A shows that during the conversion process of land use types in the Yellow River Basin of Shaanxi Province from 2000 to 2010, the conversion of cultivated land was the most severe, with a total transfer area of 3194.05 km^2^. For artificial, grass, and forest land, the transfer area was 1559.61, 989.53, and 450.16 km^2^, respectively; the conversion of grassland to other land types was more severe, with a transfer area of 1690.09 km^2^. The top three areas with the largest conversion from grassland to other land types are, cultivated land, forest, and shrubland with transfer areas of 1060.96, 307.76, and 150.49 km^2^, respectively. The transfer area of artificial surfaces is 615.90 km^2^, the main transfer type is cultivated land with an area of 589.48 km^2^, and the transfer area of forest is 433.85 km^2^. The transfer areas were sorted as follows: Cultivated land > grassland and the transfer-out areas were 324.51 km^2^ and 90.4 km^2^, respectively. Wetlands and water bodies were relatively small with transfer areas of 168.92 km^2^ and 252.12 km^2^, respectively. The total area of shrubland and bare land was small, and the conversion was not significant. Figure 4B shows the conversion of land use types from 2010 to 2020, the conversion of grassland was most severe, with a total area of 5858.58 km^2^, followed by cultivated land, forest, and artificial surfaces with transfer areas of 3155.60, 1405.16, and 565.91 km^2^ respectively. The conversion of cultivated land is frequent, and the total transferred-out area is 4922.38 km^2^. The top three areas with the largest area of cultivated land that were converted to other land types were grassland, artificial surfaces, and forest with transfer areas of 2450.65, 1583.78, and 688.29 km^2^, respectively. The total forest transfer area is 2291.98 km^2^, and the transferred land types are mainly grassland and cultivated land, with an area of 1465.67 and 719.80 km^2^, respectively. Shrubland, artificial surfaces, and bare land have relatively small transfer-out areas of 422.106, 576.124, and 524.71 km^2^, respectively. The total transfer area of wetlands was small, and the transformation was not significant.

### 3.2. Analysis of Spatial and Temporal Changes in Ecological Risks

**Figure 5:**
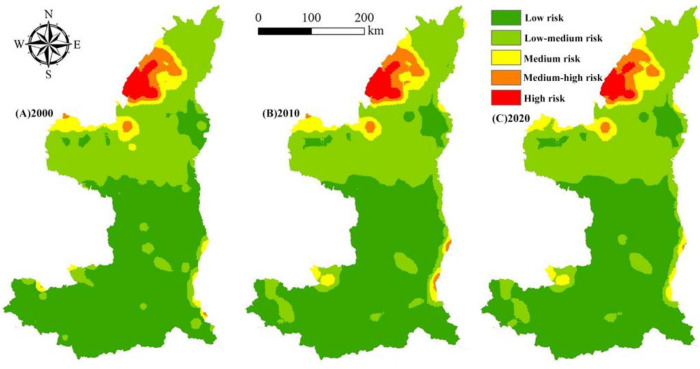
Spatial distribution changes in landscape ecological risk areas in the Yellow River Basin, Shaanxi Province from 2000 to 2020.

According to the average values and change trend of landscape ecological risk from 2000 to 2020 (Figure 5), the overall landscape ecological risk of the Shaanxi Yellow River Basin is at a lower risk level, and the temporal change showed an increasing trend at first and then a decreasing trend. From 2000 to 2010, the increase rate was relatively clear, increasing from 0.0198 to 0.0203, and the decrease rate was significant from 2010 to 2020, decreasing from 0.203 to 0.0197. The landscape ecological risk value was in a state of decline over the span of 20 years (2000–2020). Since the 18th National Congress of the Communist Party of China proposed the concept of an ecological civilization to build a beautiful China, Shaanxi Province has actively responded by implementing a series of important ecological protection and restoration projects directly impacting the Yellow River Basin ecosystem. The landscape ecological risk showed a benign development trend; thus, it is necessary to coordinate the relationship between economic construction and environmental protection in the future.

**Figure 6:**
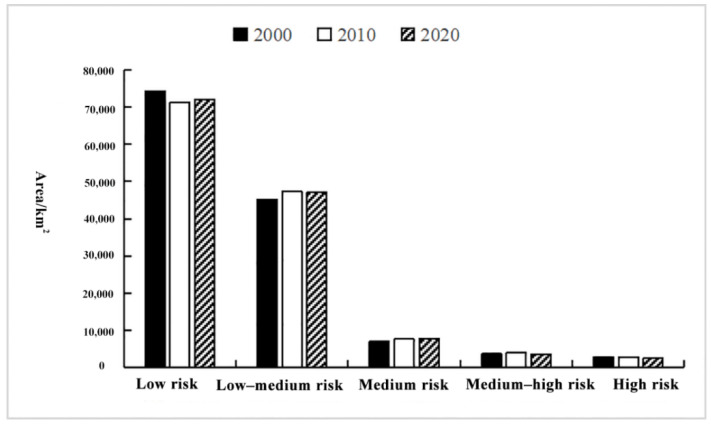
Area (km^2^) change of landscape ecological risk area of the Yellow River Basin, Shaanxi Province from 2000 to 2020.

The proportion of different ecological risk areas in the Yellow River Basin from 2000 to 2020 and the temporal change characteristics of ecological risk levels in the basin over 20 years is shown in Figure 6. These data show the area distribution of each ecological risk area and indicate that the low-risk areas are always the largest and the high-risk areas are the smallest (low-risk > medium-risk > high-risk area). For example, in 2020, the low-risk areas accounted for 54.07% of the total basin area, the low-medium-risk areas accounted for 35.35%, and the medium-risk, medium-high-risk, and high-risk areas accounted for only 5.92%, 2.77%, and 1.89%, respectively.

Considering the temporal change characteristics of the ecological risk areas over the span of 20 years, the low-risk and low-medium-risk areas have always been dominant in terms of proportion, but there are still significant changes in the ecological risk areas. The size of the low-risk areas first decreased and then increased, decreasing from 74,208.75 km^2^ in 2000 to 71,238.50 km^2^ in 2010, and increasing to 72,031.50 km^2^ in 2020. The low-risk areas showed first an increase and then a decrease. However, the increase was evident in the first 10 years, and only a slight decrease was observed from 2010 to 2020. The size of medium-risk areas increased continuously, but the increase in the first 10 years was substantially higher than that of the next 10 years. The area increased by 579.25 km^2^ in 2010 and 175.5 km^2^ in 2020. The size of the high-risk areas showed relatively clear growth from 2000 to 2010, increasing by 357.25 km^2^, and then declined from 2010 to 2020, decreasing by 398.75 km^2^. The size of the high-risk areas continued to decrease throughout the study period with a total decrease of 401.5 km^2^ over the span of 20 years.

### 3.3. Analysis of the Spatial Pattern of Ecological Risks

#### 3.3.1. Global Spatial Autocorrelation Analysis

**Figure 7:**
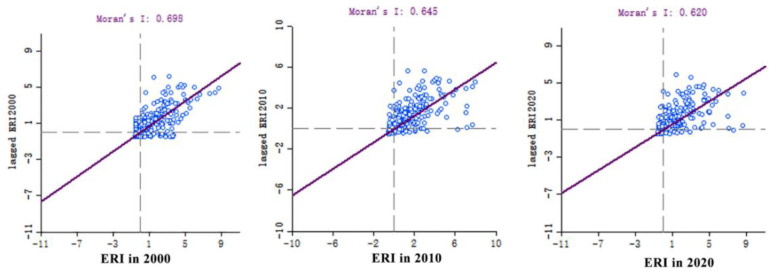
Moran’s I index scatter distribution of the landscape ecological risk values in the Shaanxi Yellow River Basin from 2000 to 2020.

According to Figure 7, the global Moran’ I index of the landscape ecological risk values from 2000, 2010, and 2020 all exceeded 0.5, and were 0.698, 0.645, and 0.620, respectively, indicating that the landscape ecological risk values in the study area were positively correlated.

In addition, the distribution of the scattered points in Figure 4 is close to the regression line, indicating that the ecological risk values of the watershed landscape have characteristics of agglomeration in the spatial distribution. Meanwhile, the z-scores of the landscape ecological risk values from 2000, 2010, and 2020 were 36.281, 33.774, and 32.515, respectively, all exceeding 1.65, indicating that the elements in the spatial distribution are non-random processes, and the possibility of a random generation of clustering patterns is unlikely. Additionally, the calculated *p*-values are all equal to 0.001, indicating that the spatial autocorrelation is significant at the 99.9% confidence level.

#### 3.3.2. Local Spatial Autocorrelation Analysis

**Figure 8:**
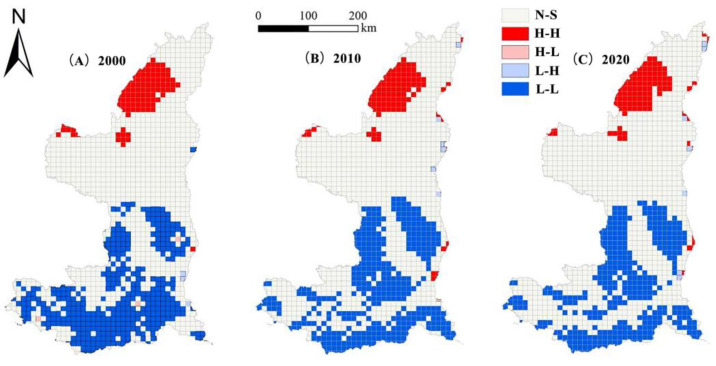
Local spatial autocorrelation distribution map of landscape ecological risks in the Yellow River Basin in Shaanxi Province from 2000 to 2020.

According to Figure 8, the correlation changes of the landscape ecological risk index are consistent with the risk distribution map of ordinary kriging interpolation of the landscape ecological risk values. The ecological risk values of the watershed landscape are mainly distributed in high-high (H-H) and low-low(L-L) agglomeration. This area belongs to the ecological protection barrier area of the Mu Us Sandy Land, which has a high degree of land loss and a fragile natural ecosystem, reducing its ability to resist risks; thus, forming a high-risk area cluster that is consistent with the distribution of low-risk areas. This area is mainly dominated by cultivated land, forest, and artificial surface land types. It is relatively flat, landscape loss is low, landscape internal structure is stable, and anti-interference ability is strong, forming a cluster of low-risk areas.

## 4. Discussion

### 4.1. Tempo-Spatial Changes of the Land Use and Landscape Ecological Risk in YRBS

Land use in YRBS experienced dramatic changes from 2000 to 2020. On the whole, the land use change was affected by human activities and natural factors. This is consistent with other research findings [63,64]. We found that cultivated land, forest land, and grassland had the largest changes and were the main land use types in the YRBS. It is closely related to the continuous promotion of ecological protection policies in Shaanxi Province. Shaanxi Province is one of the first provinces in China to pilot the policy project of returning farmland to forests and grasslands [65,66]. A series of policies of returning farmland to forests and grasslands have enabled the restoration of forest land and grassland areas, which relieved the pressure that was brought by the ecological footprint to a certain extent. In order to further improve the ecological carrying capacity of the YRBS and build a good ecological environment, it is necessary to continue to implement strict ecological environmental protection policies, and promoting the construction of ecological civilization is the key to promoting the sustainable development of the basin [67].

Our study found that high-risk areas in the YRBS are located in northern Shaanxi. This is related to the Loess Plateau region in the north, which has been studied by a large number of scholars [68,69,70]. However, the overall ecological risk management and control in northern Shaanxi has achieved preliminary results in the past 20 years. Ecological restoration projects such as forest (grass) and slope farmland improvement are closely related. A series of ecological restoration projects have adjusted the land use structure to a certain extent [71,72]. The area of cultivated land, forest land, and water area in northern Shaanxi has increased, which has improved the ecological conditions for agricultural production. However, there are still some problems of ecological degradation in the region. With the acceleration of urbanization, the demand for construction land has expanded rapidly, and human activities have exacerbated the division and occupation of cultivated land.

It is worth mentioning that we have studied the temporal and spatial evolution characteristics of ecological risks in the YRBS in the past 20 years, which can reflect the long-term trend of ecological quality in the basin to a certain extent. Overall, the distribution of risk areas is relatively stable. It can be judged that the vegetation of the YRBS has recovered significantly, and the ecological environment construction has achieved remarkable results, but the region is still a relatively fragile ecological environment [73,74]. In fragile areas, when the ecological service value is insufficient to maintain the self-circulation of the system, the ecological environment will deteriorate. In the context of global warming, extreme weather phenomena such as droughts, rainstorms, and floods have intensified, and it is difficult to maintain stable regional vegetation coverage. With economic development, the water demand for agriculture, industry, and urban domestic water increases, and the disparity between regional water supply and demand will become more prominent [75,76]. Therefore, it is necessary to strengthen the assessment of the impact of climate change on regional water resources and to improve the climate change response capabilities of key ecological function areas and ecological restoration and management areas, so as to better meet the needs of regional high-quality development.

### 4.2. Ecological Protection and High-Quality Development of the YRBS

Located in the middle reaches of the Yellow River, Shaanxi is an important national advanced manufacturing base, national defense science and technology industrial base, agricultural high-tech industrial base, energy and chemical base, and scientific, educational, and cultural base. It is the core area of ecological protection and economic and social development in Shaanxi Province [77]. The task of ecological protection is heavier. Compared with the goal of beautiful scenery in ecological space, the quality of forests in the Yellow River Basin is relatively low, the stock volume per unit area and the value of ecological service functions are lower than the national average level, the structure of forest and grass is unreasonable, the water conservation capacity is not high, the pressure of water ecological protection is high, and the area of some wetlands has shrunk [78,79]. Governance tasks remain daunting. Soil erosion control has a long way to go. The Baiyu Mountains, Weibei Dry Belt, and other key areas have a lot of historical debts, and the cost of control is high. The relationship between water and sediment is still inconsistent, and the water and sediment control system still needs to be improved. The construction of garbage and sewage treatment facilities in individual cities and towns is relatively lagging behind, and the water quality of some river sections such as the Yanhe River, Qingjian River, and Beiluo River cannot reach the standard stably. The progress of governance in coal mining subsidence areas is relatively slow. The problem of water shortage is prominent. The precipitation in the basin is low, the total water resources only account for one third of the province, and the per capita water resources are less than one fifth of the country. Xi’an, Xianyang, and other cities have serious over-exploitation of groundwater, and the ecological water volume of Yanhe, Wudinghe, Hongjiannao, and other rivers and lakes is insufficient, and some counties and districts are still seriously short of water resources, engineering, and water quality [80]. Development has caused certain constraints, and the way of water for production and living is relatively extensive. The quality of development needs to be improved urgently. The effect of implementing the new development concept is not obvious enough, the industrial structure adjustment and transformation and upgrading are relatively heavy tasks, the transformation of advantages in science, education, military industry, etc., is not sufficient, and the shortcomings of insufficient openness are still obvious. The central city’s radiating and driving role is not strong, the regional urban and rural development is unbalanced, there are still shortcomings in the field of people’s livelihood, and the modernization level of the governance system and governance capacity is not high [81,82].

According to the different regional characteristics and development orientations of the YRBS, we will coordinate the implementation of ecological governance measures that are differentiated, complete in governance elements, and reasonably and scientifically configured. Promoting the construction of check dams, changing slopes to ladders, and making land for ditch management according to local conditions. On the basis of following the laws of nature and resource endowments, implement suitable forests for forests, irrigation for irrigation, grasses for grasses, and famines for famines, and scientifically carry out land consolidation, high-standard farmland construction, and forest vegetation conservation and restoration. Coordinate the high-quality governance of mountains, rivers, forests, fields, lakes, grass, and sand and the construction of ecological projects, promote the restoration of ecosystem functions, comprehensively optimize the ecological environment of the Loess Plateau in northern Shaanxi, and consolidate the foundation for the healthy operation of the agricultural ecosystem [83]. To coordinate the relationship between ecological risk management and high-quality agricultural development, it is necessary to build a modern agricultural industrial cluster on the Loess Plateau that is based on regional ecological risks and resource endowments. Build an intensive, high-value green agricultural industry cluster that is focusing on grains (millet, barley, etc.), forest fruits (apples, red dates, etc.), edible fungi, and characteristic plants (medlar, sea buckthorn, hops, etc.), feed, meat (Beef cattle, sheep, etc.), dairy industry, cashmere-based agriculture, and animal husbandry combined with conservation agriculture and conservation grassland agriculture industry clusters. Strengthen the integration and matching of natural endowments and production factors; promote the development of regional agricultural industries to complement soil erosion control, farmland water conservancy construction, and circular agricultural projects; and support the development of eco-friendly new technologies, new formats, and new models [84].

### 4.3. Limitation

There are a few limitations and improvements that need further in-depth research and discussion: (1) Due to the difficulty of data collection, this study only started from the level of landscape structure changes that were caused by human activities as the source of risk. However, the basin is affected by natural factors such as soil erosion, meteorological changes, and geological disasters. Therefore, in the future, it is necessary to comprehensively evaluate the ecological risk from the perspective of multiple risk sources. (2) In this study, we only analyzed the temporal and spatial evolution of the existing land use data, therefore, there is no information on the future possibilities of land use in the river basin. Thus, the next step is to conduct prediction research. (3) Due to data availability and research time factors, this study only provides management suggestions that are based on the research results from the perspective of different risk levels. We did not build an indicator system for ecological risk influencing factors in detail but conducted a driving factor analysis. Generally, multi-dimensional analyses of the impact mechanism of the ecological risk in the basin are carried out.

## 5. Conclusions

The land use types in the YRBS have changed significantly over the span of 20 years. From 2000 to 2020, the overall landscape ecological risk value of the Yellow River Basin first increased then decreased, but the overall ecological risk level of the basin was lower, indicating that the ecological status of YRBS improved. The spatiotemporal evolution characteristics of five different levels of ecological risk areas in the YRBS that were discovered through this study provide scientific insights into the laws of ecological evolution in different regions in the river basin, which can further provide insights into regional ecological environment management, ecological security protection, risk early warning, and provide theoretical support for policy formulation in sustainable development and other aspects. The research conclusion can promote the sustainable development of land use and high-quality ecological protection in Changsha River beach.

In the future, further research can improve the evaluation index system of ecological risk, and not only carry out ecological risk evaluation on the basis of land use change, but also a more complex and richer index system and evaluation system should be considered. At the same time, it is also possible to analyze the driving mechanism of the evolution of ecological risks, especially considering the impact of socioeconomic factors and human activities on ecological risks.

## Figures and Tables

**Table 1:** Calculation formula of landscape index and ecological significance.

Index	Calculation Formula	Ecological Significance
Landscape loss degree index (*R_i_*)	$R_{i}=E_{i}\times F_{i}$	*R_i_* indicates the degree of loss of natural properties of ecosystems represented by different landscape types when they are subjected to natural and anthropogenic disturbances [54].
Landscape disturbance index (*E_i_*)	$E_{i}=aC_{i}+bN_{i}+cD_{i}$	*E_i_* describes the extent to which ecosystems located in different landscape types are disturbed by human activities and characterizes differences related to maintenance of ecological stability of different landscape types [57]; a, b, and c represent weights of the corresponding landscape indices; according to results of previous studies, values of a = 0.5, b = 0.3, and c = 0.2 are assigned.
Landscape fragmentation index (*C_i_*)	$C_{i}=\frac{n_{i}}{A_{i}}$	Describes the degree of fragmentation of a landscape type in the region at a given time; such that, the higher its value, the lower the stability within the landscape unit and the greater the heterogeneity and discontinuity among patches [58]; *n_i_* denotes the number of patches of landscape type *i* and *A_i_* denotes the total area of landscape type *i*.
Landscape dominance index (*D_i_*)	$D_{i}=\frac{Q_{i}+M_{i}}{4}+\frac{L_{i}}{2}$	The higher the value, the greater the influence of the landscape type on the overall landscape pattern [59]. *Q_i_* = number of samples in which patch *i* occurs/total number of samples; *M_i_* = number of patch *i*/total number of patches; and *L_i_* = area of patch *i*/total area of samples.
landscape separateness index (*N_i_*)	$N_{i}=\frac{A}{2A_{i}}\sqrt{\frac{n_{i}}{A}}$	The greater the degree of separation between different patches in a landscape type, the more discrete the distribution of the landscape type in the region for a correspondingly higher degree of fragmentation [60]; A is the total area of the landscape; *N_i_* is the distance index of landscape type *i*.
Landscape vulnerability index (*F_i_*)	Based on the previous studies	The higher the value, the more vulnerable and unstable the landscape type is and the more likely it will suffer ecological losses and physical changes due to external disturbances [61]. Based on the previous studies, in this study [62], vulnerability indices of six landscape types were assigned as follows: unused land 6, water 5, cultivated land 4, grassland 3, woodland 2, and residential land 1, with the landscape vulnerability index *F_i_* obtained after normalization.

**Table 2:** Landscape ecological risk classification in the Yellow River Basin, Shaanxi Province, China.

Ecological Risk	Risk Level				
	Low	Low-Medium	Medium	Medium-High	High
rank	I	II	III	IV	V
value	0.0135 < ERI	0.0135 ≤ ERI < 0.030	0.030 ≤ ERI < 0.060	0.060 ≤ ERI < 0.099	ERI ≥ 0.099

**Table 3:** Change in the area and single dynamic degree of land types in the Yellow River Basin, Shaanxi Province, China.

Land Type	2000–2010		2010–2020		2000–2020	
	Area Change (km^2^)	Single Dynamics (%)	Area Change (km^2^)	Single Dynamics (%)	Area Change (km^2^)	Single Dynamics (%)
Cultivated Land	−1000.56	−0.17	−197.86	−0.03	−1198.41	−0.11
Forest Land	351.25	0.09	−118.57	−0.03	232.68	0.03
Grassland	−502.24	−0.11	−1232.16	−0.28	−1734.4	−0.2
Shrubland	159.93	3.14	−28.45	−0.41	131.48	1.36
Wetland	−54.8	−2.03	−10.77	−0.51	−65.56	−1.28
Water Body	−40.31	−0.69	30.08	0.56	−10.23	−0.09
Artificial Surface	1072.16	3.11	1665.78	3.59	2737.94	4.21
Bare Ground	14.47	0.09	−103.61	−0.64	−89.13	−0.29
Comprehensive Dynamics (%)	0.108		0.114		0.111	

## Data Availability

The data that support the findings of this study are available from the corresponding author upon reasonable request.
